# Efficacy of the Combined Protective Cultures of *Penicillium chrysogenum* and *Debaryomyces hansenii* for the Control of Ochratoxin A Hazard in Dry-Cured Ham

**DOI:** 10.3390/toxins11120710

**Published:** 2019-12-05

**Authors:** Eva Cebrián, Mar Rodríguez, Belén Peromingo, Elena Bermúdez, Félix Núñez

**Affiliations:** Food Hygiene and Safety, Meat and Meat Products Research Institute, Faculty of Veterinary Science, University of Extremadura, Avda. de las Ciencias, s/n, 10003 Cáceres, Spain; evcebrianc@unex.es (E.C.); belenperomingo@unex.es (B.P.); bermudez@unex.es (E.B.); fnunez@unex.es (F.N.)

**Keywords:** *Penicillium nordicum*, biocontrol agents, dry-cured ham, ochratoxin A (OTA)

## Abstract

The ecological conditions during the ripening of dry-cured ham favour the development of moulds on its surface, being frequently the presence of *Penicillium nordicum*, a producer of ochratoxin A (OTA). Biocontrol using moulds and yeasts usually found in dry-cured ham is a promising strategy to minimize this hazard. The aim of this work is to evaluate the effect of previously selected *Debaryomyces hansenii* and *Penicillium chrysogenum* strains on growth, OTA production, and relative expression of genes involved in the OTA biosynthesis by *P. nordicum*. *P. nordicum* was inoculated against the protective cultures individually and combined on dry-cured ham for 21 days at 20 °C. None of the treatments reduced the growth of *P. nordicum*, but all of them decreased OTA concentration. The lower production of OTA could be related to significant repression of the relative expression of *otapks*PN and *otanps*PN genes of *P. nordicum*. The efficacy of the combined protective cultures was tested in 24 dry-cured hams in industrial ripening (an 8 month-long production). OTA was detected in nine of the 12 dry-cured hams in the batch inoculated only with *P. nordicum.* However, in the batch inoculated with both *P. nordicum* and the combined protective culture, a considerable reduction of OTA contamination was observed. In conclusion, although the efficacy of individual use *P. chrysogenum* is great, the combination with *D. hansenii* enhances its antifungal activity and could be proposed as a mixed protective culture to control the hazard of the presence of OTA in dry-cured ham.

## 1. Introduction

The ecological conditions reached during the ripening of dry-cured ham favour the growth of moulds on their surface, mainly belonging to the genera *Penicillium* and *Aspergillus* [1,2]. The presence of moulds is valuable due to their contribution to proteolytic and lipolytic changes observed during the ripening [3], and consequently, to the development of required sensory characteristics of dry-cured ham [4,5]. However, most of the moulds isolated in these products are potentially toxigenic [1,6,7].

Ochratoxin A (OTA) is the mycotoxin most commonly found in meat products, which contribute significantly to human exposure to this toxin [8,9]. OTA has nephrotoxic, immunosuppressive, genotoxic, carcinogenic, teratogenic and neurotoxic effects [10,11] and, it has been classified by the International Agency for Research on Cancer (IARC) as a possible human carcinogen in the 2B category [12].

The presence of OTA in dry-cured ham could be a consequence of the growth of *Aspergillus westerdijkiae* [13], *Penicillium verrucosum* and *Penicillium nordicum* [2,14,15,16]. Among them, *P. nordicum* is the most common ochratoxigenic mould isolated from dry-cured meats [2,15,17], due to its special adaptation to protein and NaCl-rich foods [2,16,18,19].

Due to the risk to human health, the European Union has set the maximum level permissible of OTA for different foodstuffs such as cereals, wine, fruits, coffee or baby foods, although not in meat products [20]. In addition, Italy has established the maximum guide value for OTA in pork and pork products at 1 μg/kg [21]. Therefore, it is important to implement strategies to control the growth of ochratoxigenic moulds and the production of this mycotoxin. 

There are several effective antifungal treatments, including physical methods and chemical preservatives, but most of them are not adequate for mould-ripened foods, such as dry-cured ham, since they are non-selective and can interfere with the ripening, altering the sensory characteristics [22]. Thus, the use of microorganisms usually found in this type of foods as bioprotective agents has been proposed as a promising strategy to control toxigenic moulds in dry-cured meat products [23,24,25,26,27,28,29,30,31,32].

Yeasts are interesting as antagonistic microorganisms since they are part of the natural microbiota of dry-cured meat products, are phenotypically adapted to this environment and contribute to the development of sensory characteristics [4,33,34,35]. *Debaryomyces hansenii* is the predominant yeast species throughout the processing of dry-cured meat products [35,36], and some autochthonous strains have been proposed as biological control of OTA in these foods [24,27,28,29,30,31,32,37]. In this sense, *D. hansenii* FHSCC 253H isolated from dry-cured ham is able to reduce the production of OTA by *P. verrucosum* on this food [30]. Moreover, the species *D. hansenii* has been included in the European Qualified Presumption of Safety (QPS) list as a safe microorganism.

On the other hand, non-toxigenic moulds producers of antifungal proteins could be considered for their use as protective cultures in dry-cured meat products [38]. These antifungal proteins probably give producer moulds a selective advantage to compete with other moulds in its ecological niche [38]. In this sense, *Penicillium chrysogenum* CECT 20922, isolated from dry-cured ham and producer of the antifungal protein PgAFP [39], is able to reduce OTA contamination in dry-cured ham [40]. The protective effect of *D. hansenii* against OTA-producer moulds in meat substrates has been attributed to a combination of competition for space and nutrients, production of soluble metabolites [28], and interfering the secondary metabolism [41], mainly by the reduction relative expression of the OTA biosynthetic genes [30]. Meanwhile, the preventive activity of *P. chrysogenum* has been linked to the production of antifungal protein PgAFP [40], nutrient competition and hampering the secondary metabolism of *P. nordicum* [41]. Then, the combined use of these bicontrol agents on dry-cured ham could have an additive or synergistic effect against the growth of *P. nordicum* and OTA contamination, but this fact has not been demonstrated yet. Therefore, it is necessary to evaluate the implantation of these potential protective microorganisms in combined cultures in the presence of toxigenic moulds in dry-cured ham during the ripening process. In addition, the effect of these biocontrol agents over toxigenic mould growth and accumulation of mycotoxins should be also evaluated. 

Although the biosynthetic pathway of OTA has not been fully clarified, two genes have been described as required by *P. nordicum* for the biosynthesis of OTA, the polyketide synthetase gene (*otapks*PN), and the non-ribosomal peptide synthetase gene (*otanps*PN) [42]. Therefore, these two genes can be used as a target for the study of gene expression in *P. nordicum* [43]. Finally, among the genes of interest for endogenous control is the gene *β-tubulin* that encodes a globular protein that is the main component of the microtubules of fungal cells [7,44].

The main objective of the present work is to evaluate the effect of *D. hansenii* FHSCC 253H and *P. chrysogenum* CECT 20922 as a combined protective culture in the growth of *P. nordicum* FHSCC IB4, the OTA production and the expression of genes involved in the biosynthetic pathway of this mycotoxin in dry-cured ham.

## 2. Results

### 2.1. Effect of Bioprotective Agents on P. nordicum Growth in Dry-Cured Ham

The growth of toxigenic mould and bioprotective agents inoculated in dry-cured ham was evaluated by plate counting in MEA. 

All microorganisms were able to grow properly on dry-cured ham, reaching counts higher than 10^6^ cfu/cm^2^ at the end of incubation in each batch. While the presence of each bioprotective agents separately did not affect *P. nordicum* counts, their use in combination produced a significant reduction (*p* ≤ 0.01) in the growth of the toxigenic mould (Table 1).

### 2.2. Influence of Bioprotective Agents on OTA Production by P. nordicum

Table 2 shows the effect of the bioprotective agents on the production of OTA by *P. nordicum* in dry-cured ham after 21 days of incubation at 20 °C.

The amount of OTA was significantly reduced (*p* ≤ 0.05) when *P. nordicum* was inoculated with any of the bioprotective cultures individually as well as it was inoculated with both combined. The lower concentration of OTA was observed in the batches when *P. chrysogenum* was inoculated combined with *D. hansenii* (98.5%). Although no significant differences were detected between the individually inoculated batches, there was a greater reduction in the mean of OTA amount in the batch inoculated with *P. chrysogenum* (97.2%) than with *D. hanseni* (82%).

### 2.3. Effect of Bioprotective Agents in the Relative Expression of otapksPN and otanpsPN Genes in P. nordicum

**Figure 1:**
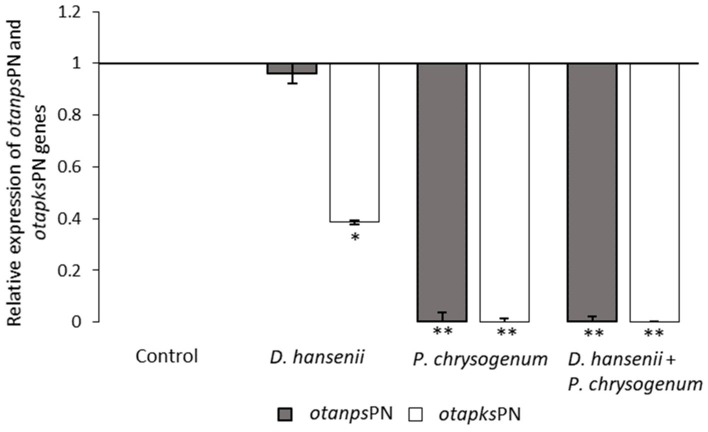
Relative expression of the *otanps*PN and *otapks*PN genes in *P. nordicum* inoculated in dry-cured ham without bioprotective cultures (control) with *D. hansenii* and *P. chrysogenum* individually, or both combined after incubation for 21 days at 20 °C. Significant differences of the gene expression of each batch compared to control are indicated by asterisk: * *p* ≤ 0.05; ** *p* ≤ 0.01.

The effect of bioprotective agents on the biosynthesis of OTA was assessed by studying the relative expression of the *otanps*PN and *otapks*PN genes by *P. nordicum* over a 21-day incubation period in dry-cured ham (Figure 1). The threshold cycle (Ct) values of *β-tubulin* genes detected in qPCR analyses were always between 21 and 26 after 21 days of incubation, which is indicative that the moulds continued in active growth. The relative expression of the *otapks*PN genes was significantly reduced by both tested microorganisms. For *otanps*PN a reduction of the expression was observed in the batches inoculated with *P. chrysogenum*. In the same way as in the production of OTA, the highest inhibition level of both gene expressions was observed when *P. chrysogenum* was inoculated individually or in combination with *D. hansenii*.

### 2.4. Effect of Combined Protective Cultures on OTA Contamination in Dry-Cured Ham Inoculated with P. nordicum in Small Scale Manufacture

Table 3 shows the results of OTA concentration at the end of the processing of the 24 dry-cured hams. In the control batch, 9 of 12 hams were contaminated with OTA, and 6 of them showed OTA levels ranged from 7.71 to 1620 μg/kg, above the guide value permitted for pork meat and derived products in Italy [21]. However, the amount of OTA was substantially lower in the batch inoculated with the combined bioprotective culture. In this batch, 8 of 12 samples OTA was no detected (<0.58 ppb), and the 4 OTA-contaminated hams did not exceed levels of 11.73 μg/kg. 

## 3. Discussion

In this work, the use of *D. hansenii* and *P. chrysogenum* strains isolated from dry-cured ham as biocontrol agents were evaluated. Although both strains used had proved their ability to reduce the concentration of OTA on dry-cured meat products individually, their potential synergistic effect had not yet been tested.

Both bioprotective agents, whether inoculated individually or combined, grew satisfactorily in the presence of *P. nordicum* on dry-cured ham in environmental conditions similar to those reached during the usual processing (Table 1). However, despite this strong growth, only when the bioprotective agents were used in combination, a statistically significant decrease in *P. nordicum* counts was observed. However, even in the batch inoculated with *D. hansenii* and *P. chrysogenum*, the count reached by *P. nordicum* was above the level estimated to be required for the accumulation of OTA in dry-cured ham [15]. This same strain of *D. hansenii* also failed to decrease the growth of the *P. verrucosum* producer of OTA in dry-cured ham incubated in similar conditions to those described in the present work [30]. However, in different culture media, this strain was able to delay the germination of *P. nordicum* spores [24] and decrease the growth rate of *P. verrucosum* [30] in the range of 0.90–0.97 *a_w_*. This antifungal activity has been linked mainly to the competition for nutrients and the production of yeast metabolites [28]. The discrepancies in the antifungal activity of *D. hansenii* between that studies can likely be attributed to the different moulds tested [28] and the diverse environmental conditions, such as substrate or *a_w_* [24,31].

Regarding *P. chrysogenum* CECT 20922, its inoculation did not have any noticeable influence on the total count of ochratoxigenic moulds observed throughout the ripening of dry-cured hams [40]. On the contrary, this *P. chrysogenum* strain reduced the levels of aflatoxigenic *Aspergillus flavus* in dry-cured ham [45] to non-detectable levels. Given that the antagonistic activity of *P. chrysogenum* CECT 20922 has been related with the production of the antifungal protein PgAFP [40], the differences in efficiency against different moulds in a similar substrate may be due to the greater sensitivity of *A. flavus* than OTA-producing moulds, such as *P. nordicum* and *P. verrucosum*, to PgAFP [46].

Concerning the production of OTA by *P. nordicum*, both bioprotective agents tested, used individually or combined, were effective in decreasing the concentration of this mycotoxin. However, in the batch inoculated with *D. hansenii,* the reduction of OTA amount is insufficient to meet the Italian guideline value of 1 μg/kg for pork derived products [21]. The maximum reduction was obtained with *P. chrysogenum* alone or in combination with *D. hansenii*. In these batches, the concentration of OTA is lower than 1 μg/kg, and they may be regarded as safe according to Italian regulation [21]. In addition, the study carried out in the dry-cured hams confirms the antifungal effect of the combined culture of *P. chrysogenum* and *D. hansenii*. In the batch inoculated with both protective microorganisms, OTA was detected just in 33% of dry-cured hams, whereas in the batch inoculated only with *P. nordicum*, 75% of hams were found to contain OTA (Table 3). In addition, in the four contaminated hams treated with protective cultures, the average level of OTA (7.89 µg/kg) was considerably lower than that of the nine contaminated hams from control batch inoculated only with *P. nordicum* (510.31 µg/kg). The differences found between the samples from the control batch can be attributed to the high heterogeneity in mould growth and mycotoxin distribution among different parts of solid foods. Since moulds often grow in certain spots on the food surface, it can be detected the presence of high contamination levels in a relatively confined or small part of a food [47]. 

Different strains of *D. hansenii* have demonstrated their ability to significantly decrease the production of OTA by *A. westerdijkiae* [37] and *P. nordicum* [32] in standard culture media and to completely inhibit the production of OTA by *P. nordicum* and *A. ochraceus* in dry-cured ham without altering its organoleptic properties [27]. In addition, the strain *D. hansenii* FHSCC 253H used in this work has previously shown its efficacy in reducing the production of OTA of *P. verrucosum* and *P. nordicum* [24,30] and aflatoxins B1 and G1 by *Aspergillus parasiticus* [29] in dry-fermented sausages and dry-cured ham.

In the same way, some non-toxigenic moulds have shown their capability to significantly decrease the presence of OTA in dry-cured meat products processed in meat industries, including *Penicillium nalgiovense* [48] and the strain of *P. chrysogenum* used in this work [40].

Although mould growth should not be considered the only parameter to predict OTA contamination in foods [16,49], a positive correlation has been described between the growth of ochratoxigenic moulds and the production of OTA on meat substrates [30]. However, considering the limited effect observed on the growth of *P. nordicum* (Table 1), it can be inferred that the recorded drastic decrease in OTA (Table 2) is not due to the influence of bioprotective agents on toxigenic mould development. 

Among the mechanisms of action of yeasts to decrease the content of mycotoxins, the enzymatic degradation, the adsorption to the cell wall or the repression of the biosynthesis route has been described [22]. However, the *D. hansenii* strain tested does not degrade or adsorb OTA nor aflatoxins, but reduces the synthesis of these toxins by *P. verrucosum* [30] and *A. parasiticus* [29], respectively, in meat substrates. As in the present study, this reduction occurs without growth inhibition, and it was attributed to a blockage in the expression of genes related to the synthesis of mycotoxins [29,30]. The blockage of OTA-related genes was described in culture media against *P. verrucosum* [30], but this mechanism has not been proved with *P. nordicum* in dry-cured ham.

Then, to confirm this mechanism of action involved in the reduction of OTA in dry-cured ham, the effect of the protective agents on the expression of the *otapks*PN and *otanps*PN genes involved in the OTA biosynthetic pathway was evaluated. The expression of these two genes was repressed when *D. hansenii* and *P. chrysogenum* were inoculated both individually and combined against *P. nordicum*. In relation to *D. hansenii*, the *otapks*PN gene was more repressed than the *otanps*PN gene, obtaining 61% and 4% repression, respectively. This repression is consistent with studies that demonstrated that *D. hansenii* is able to reduce expression of the *pks* and *p450-B03* genes involved in the OTA production by *A. westerdijkiae* [37] and to inhibit the expression of the *otanps*PN gene of *P. verrucosum* in meat-based culture media [30].

With respect to *P. chrysogenum*, the repression was very similar in both *otapks* (99.99%) and *otanps* genes (99.62%). This repression could be complementary to the inhibition of the carbon catabolite repression (CCR) pathway as the consequence of the nutritional competition between *P. nordicum* and *P. chrysogenum* in meat substrates, evidenced by proteomic analysis [41]. This CCR pathway has been linked to secondary metabolism and with mycotoxins production [50]. These mechanisms may explain the effect of *P. chrysogenum* decreasing the OTA production by *P. nordicum* without affect the growth. However, *P. nordicum* showed the capacity to grow when it was co-inoculated with the bioprotective agents used in this work on dry-cured ham under the environmental conditions usually found through the ripening of this product. Nevertheless, the real hazard of *P. nordicum* is the production of OTA, and this was reduced in the presence of *D. hansenii* and *P. chrysogenum* used either individually or combined. This reduction is related to the repression of the *otapks*PN and *otanps*PN genes involved in the biosynthetic pathway of this mycotoxin. In addition, the efficacy of the bioprotective agents against the production of OTA by *P. nordicum*, on dry-cured hams processed in the usual environmental conditions set in the meat industries has been demonstrated. In conclusion, the use of *D. hansenii* FHSCC 253H and *P. chrysogenum* CECT 20922 could be proposed as an innovative combined protective culture for the prevention of OTA hazard in dry-cured ham.

## 4. Materials and Methods

### 4.1. Microorganisms

In this study the OTA-producer *P. nordicum* FHSCC IB4, the antifungal *D. hansenii* FHSCC 253H, both from the the Food Hygiene and Safety Culture Collection at the University of Extremadura (Cáceres, Spain), and the PgAFP-producer *P. chrysogenum* CECT 20922, from the Spanish Type Culture Collection (Valencia, Spain) were used. All three strains were isolated from dry-cured ham [16,28,38]. The ability of *P. nordicum* to produce OTA [16] and the antifungal activity of *D. hansenii* [24,28,29,30] and *P. chrysogenum* [40,41] have been previously assessed.

### 4.2. Preparation of Mould and Yeast Inocula

*P. nordicum* and *P. chrysogenum* were grown on potato dextrose agar (PDA) (Scharlab S.L., Sentmenat, Spain) at 25 °C for 7 days. Conidia were collected by adding 4 mL of saline phosphate buffer (PBS) and scraping the surface with a sterile glass rod. The spores were quantified by using a Thoma counting chamber Blaubrand^®^ (Brand, Bremen, Germany), and adjusted to 10^5^ spores/mL and used as inoculum.

The inocula of *D. hansenii* was obtained by inoculating 100 μL from a culture preserved at −80 °C in YES broth (Scharlab S.L., Sentmenat, Spain). It was incubated for 72 h at 150 rpm and 25 °C. The culture was centrifuged at 3500 rpm for 5 min, and the concentrated cells were resuspended in PBS. The yeast suspensions were counted by using the Thoma chamber and adjusted to 10^6^ yeasts/mL to be used as inoculum.

### 4.3. Preparation of the Dry-Cured Ham Portions

Pieces of dry-cured ham were cut into cubes of approximately 3 × 3 × 3 cm. To reduce microbial contamination of pieces, they were firstly submerged in ethanol for 30 s, and the surface was disinfected in a laminar flow hood with ultraviolet light for 2 h. After this time, each of the blocks was immersed in sterile distilled water for few seconds to be rehydrated. Then, the *a_w_* of pieces was 0.88.

### 4.4. Experimental Setting

Four different batches were prepared: the control batch, inoculated only with *P. nordicum*, one batch inoculated with *P. nordicum* and *D. hansenii*¸ one batch inoculated with *P. nordicum* and *P. chrysogenum*, and one batch inoculated with *P. nordicum*, *D. hansenii* and *P. chrysogenum*.

Fifty µL of each microorganism was inoculated on the surface of dry-cured ham using a Drigalski spreader previously sterilized. When the piece of ham was inoculated with several microorganisms, we waited 10 min between each strain inoculation to allow the absorption.

The inoculated dry-cured ham cubes were incubated at 20 °C for 21 days in plastic containers previously sterilized. To simulate the dehydration that occurs during the ripening of dry-cured ham, a relative humidity of 94% was maintained inside the container by depositing 400 mL of oversaturated K_2_SO_4_ solution at the bottom of the containers. The experiment was carried out in quintupled.

### 4.5. Evaluation of Mould and Yeast Growth

At the end of incubation time, an area of approximately 5 cm^2^ of the surface of each dry-cured ham piece was homogenized with 45 mL of 0.1% peptone water (Panreac Quimica S.L.U., Barcelona, Spain) in a filter bag BagPage (Interscience, Saint Nom, France) using a Stomacher (Stomacher^®^ 400 Circulator, Seward, Worthing, UK). After performing decimal dilutions, the growth of the inoculated microorganisms was determined by plating in malt extract agar (MEA, Scharlab S.L., Sentmenat, Spain), and incubating at 25 °C for 7 days. The colonies of each strain were distinguished by their morphology, even the two species of mould presented clear differences in colour and size. *P. nordicum* showed a whitish colour with smaller colonies, while *P. chrysogenum* colonies were green and larger. The sampling was carried out in duplicate. 

### 4.6. Extraction and Quantification of Ochratoxin A

#### 4.6.1. OTA Extraction

OTA was extracted following the QueChERS methodology [51] with modifications. Briefly, 3 g from the surface of each dry-cured ham cube was mixed with 2 mL of water acidified with 0.1% acetic acid and shaken for 30 s. Then, 2 mL acetonitrile acidified with 0.1% acetic acid was added and shaken for 1 min. Thereupon, 0.4 g NaCl and 1.6 g MgSO_4_ were added and shaken manually for 30 s. The samples were centrifuged for 5 min at 5000 rpm, and an aliquot of 1 mL supernatant was filtered through a 0.45 μm pore size nylon membrane (MSI, Omaha, NE, USA), placed in a vial and analyzed by HPLC-FLD.

#### 4.6.2. OTA Quantification

OTA quantification was performed in Agilent 1260 Infinity (Agilent Technologies, Santa Clara, CA, USA) equipment coupled to a fluorescence (FLD) detector (Agilent Technologies, Santa Clara, CA, USA). The column Zorbax SB C18 (2.1 mm × 50 cm × 1.8 µm; Agilent Technologies, Santa Clara, CA, USA) was used. The mobile phase was acetonitrile:water:acetic acid (50:100:1 *v/v/v*) with a flow rate of 0.1 mL/min. FLD detection was performed using 330 and 450 nm excitation and emission wavelengths, respectively. The calibration curve of OTA by HPLC-FLD revealed a linear relationship (*r^2^* ≥ 0.99) between detector response and OTA standard (Sigma-Aldrich Co., San Luis, MO, USA) amounts between 0.5 and 50 ng/mL. The limit of detection (LOD) obtained in this study was 0.58 µg/kg, and the quantification limit (LOQ) was 1.94 µg/kg.

### 4.7. Relative Gene Expression

#### 4.7.1. RNA Extraction

The mycelia were removed from half the surface of the dry-cured ham cubes and frozen quickly in liquid nitrogen, it was stored at −80 °C until the extraction of RNA. RNA was extracted using the commercial kit RNeasy^®^ plant mini kit (QIAGEN, Madrid, Spain) [52]. For this, the mycelia were mixed with 500 μL of lysis solution buffer containing 10 μL of β-mercaptoethanol and then processed according to the manufacturer’s instructions. Once the process was completed, the RNA obtained was diluted in 50 μL of RNAse-free water. The RNA samples obtained were treated with the commercial kit DNase I, RNase-free (Thermo Fisher Scientific, Waltham, MA, USA), to eliminate the possible residual genomic DNA. Finally, the concentration and purity (A_260_/A_280_ ratio) of the RNA obtained was measured in a NanoDrop 2000c spectrophotometer (Thermo Fisher Scientific, Waltham, MA, USA).

#### 4.7.2. Reverse Transcription Reaction

For the synthesis of the cDNA from the RNA obtained the commercial kit "PrimeScript^TM^ RT Reagent" (Takara Bio Inc., Kusatsu, Japan) was used following the manufacturer’s indications. The cDNA synthesis reaction was performed on a Mastercycler thermocycler (Eppendorf AG, Hamburg, Germany) using the following conditions: 15 min at 37 °C; 5 s at 85 °C; and finally, a cooling to 4 °C. The resulting cDNA was stored at −20 °C until use.

#### 4.7.3. RT-qPCR

##### Primers Used

Two primers (Table 4) were used to amplify the *β-tubulin* gene, which was used as an endogenous control [52]. For the relative expression of the genes *otapks*PN and *otanps*PN involved in the biosynthesis of OTA, two pairs of primers were used [19,52].

##### RT-qPCR Methods

The qPCR was carried out in the Applied Biosystems 7500 Fast real-time PCR system (Applied Biosystems, Waltham, MA, USA) and qPCR methods based on the SYBR Green methodology were used [45,53]. They were prepared in triplicates in MicroAmp optical 96-well plates which a total volume of 12.5 µL was added to each of them, consisting of 6.25 µl 2× SYBR^®^ Premix Ex Taq^TM^, 0.5 μL of 50 ROX^TM^ reference dye, 2.5 μL of cDNA and 300 nM of each primer for the *otapks*PN, *otanps*PN and *β-tubulin* gene. In addition, three negative controls were incorporated without the target cDNA sequence. Finally, the plates were sealed with optical adhesive covers (Applied Biosystems, Waltham, MA, USA). The amplification conditions used for the qPCR reactions were: 1 cycle at 95 °C for 10 min, 40 cycles at 95 °C for 15 s and 60 °C for 1 min. After the final PCR cycle, the melting curve analysis of the PCR products was performed by heating to 60–95 °C and continuous measurement of the fluorescence to verify the PCR product.

The relative quantification of the expression of the *otapks*PN and *otanps*PN genes was performed using the housekeeping *β-tubulin* gene as an endogenous control to normalize the quantification of the mRNA target for differences in the amount of total cDNA added to each reaction. The expression ratio was calculated using the 2^−ΔΔCT^ method [54]. The value of the relative expression of the control batch inoculated with *P. nordicum* without the presence of the other microorganisms was used as a calibrator.

### 4.8. Effect of Combined Protective Culture on OTA Contamination During Ripening of Dry-Cured Ham in Small Scale Manufacture.

The protective effect of the combined biocontrol agents *P. chrysogenum* and *D. hansenii* was tested in dry-cured ham ripened under the environmental conditions usually set in industrial ripening (an 8 month-long production).

A total of 24 dry-cured hams in the final post-salting stage (2 months of ripening approximately) were divided in two batches: 12 dry-cured hams were inoculated with only *P. nordicum* (control batch), and the other 12 dry-cured hams were inoculated with *P. nordicum* and the combined protective culture (*P. chrysogenum* and *D. hansenii)*.

The inocula of both moulds and *D. hansenii* were prepared in the same way as in Section 4.2.

For the inoculation, each piece was immersed for 30 s in 25 liters solution with *P. nordicum* for the control batch, and with *P. nordicum, P. chrysogenum* and *D. hansenii* for the other batch. The dry-cured hams were hung up and ripened in two different maturation chambers for 6 months. During the first month, the temperature progressively increased from 4 to 15 °C and the relative humidity (RH) decreased from 80% to 75%. From the second to sixth month of ripening, the temperature was gently increased from 15 to 22 °C, and the RH was lowered below 70% at the end of the maturing. At the end of the processing, the dry-cured hams lost approximately 35% of their initial weight.

#### Sampling, Extraction and Quantification of OTA

Sampling was done at the end of ripening (an 8 month-long production). For that purpose, 25 cm^2^ surface areas showing fungal growth and 1 cm of depth were obtained of each dry-cured ham. The procedure of extraction and quantification of OTA was carried out as described above in Section 4.6.

### 4.9. Statistical Analysis

All data were analyzed by IBM SPSS v.22 (Chicago, IL, USA, 2013). The data normality test was performed by Shapiro–Wilks. All data sets failed the normality test; a variable transformation was performed to improve normality or homogenize the variances. Without success, the analysis of non-parametric data was performed using the Kruskal–Wallis test to determine significant differences between the means. Subsequently, those samples that differed had the U Mann–Whitney test applied to compare the mean values obtained. Statistical significance was set at *p* ≤ 0.05.

## Figures and Tables

**Table 1:** Growth of *P. nordicum*, *P. chrysogenum* and *D. hansenii* (log cfu/cm^2^) in dry-cured ham after 21 days at 20 °C.

Batches	*P. nordicum*	*P. chrysogenum*	*D. hansenii*
Control	7.02 ± 0.14	-	-
*D. hansenii*	7.41 ± 0.38	-	8.28 ± 0.18
*P. chrysogenum*	7.25 ± 0.42	6.50 ± 0.18	-
*D. hansenii* + *P. chrysogenum*	6.71 ± 0.36*	7.19 ± 0.26	8.36 ± 0.03

-: growth < 1 log cfu/cm^2^.* Significant differences with respect to control (*p* ≤ 0.01).

**Table 2:** Effect of *D. hansenii* and *P. chrysogenum* on OTA production by *P. nordicum* in dry-cured ham after 21 days at 20 °C.

Batches	OTA Concentration (µg/kg)*
Control	15.48 ± 8.87^a^
*D. hansenii*	2.78 ± 2.18^b^
*P. chrysogenum*	0.44 ± 0.63^bc^
*D. hansenii + P. chrysogenum*	0.23 ± 0.52^c^

* Values of OTA concentration followed by different letters are significantly different (*p* < 0.05).

**Table 3:** Levels of ochratoxin A (OTA) detected in samples of dry-cured ham.

Batches	Sample Reference of Dry-Cured Ham	Average OTA Concentration (µg/kg)
*P. nordicum*	1	0.39
	2	7.71
	3	1226.92
	4	-
	5	1275.55
	6	176.86
	7	-
	8	-
	9	0.58
	10	284.13
	11	1620.00
	12	0.67
*P. nordicum* + *P. chrysogenum* + *D. hansenii*	13	11.73
	14	3.67
	15	-
	16	-
	17	9.68
	18	-
	19	-
	20	-
	21	-
	22	-
	23	-
	24	6.48

-: levels less than the limit of detection.

**Table 4:** Oligonucleotide sequences of primers used in this study.

Genes	Primers	Nucleotide Sequences (5’-3’)	Product Size (pb)	Positions
*β-tubulin*	β-tubF1	GCCAGCGGTGACAAGTACGT	93	279 ^a^
	β-tubR1	TACCGGGCTCCAAATCGA		54 ^a^
*otapks*PN	otapksF3	CGCCGCTGCGGTTACT	80	1816 ^b^
	otapksR3	GGTAACAATCAACGCTCCCTCTT		1873 ^b^
*otanps*PN	F-npstr	GCCGCCCTCTGTCATTCCAAG	113	5090 ^b^
	R-npstr	GCCATCTCCAAACTCAAGCGTG		5181 ^b^

^a^ Positions are in accordance with the published sequence of *β-tubulin* gene of *P. nordicum* (GenBank accession no. AY674319.1). ^b^ Positions are in accordance with the published sequence of the *otapks* and *otanps* genes of *P. nordicum* (GenBank accession no. AY557343.2).
